# A comparison of two mail-based strategies to recruit older cancer survivors into a randomized controlled trial of a lifestyle intervention

**DOI:** 10.1007/s11764-021-01091-x

**Published:** 2021-08-03

**Authors:** Amelia C. Warnock, Harleen Kaur, J. Ryan Buckman, Teri Hoenemeyer, Wendy Demark-Wahnefried

**Affiliations:** Department of Nutrition Sciences, University of Alabama at Birmingham (UAB), Birmingham, USA

**Keywords:** Cancer survivors, Clinical trials, Lifestyle, Recruitment, Enrollment

## Abstract

**Purpose:**

To compare response rates to business letter versus greeting card invitations used to recruit older cancer survivors to a randomized controlled clinical trial of a lifestyle intervention

**Methods:**

Capitalizing upon recruitment efforts for a lifestyle intervention trial among older cancer survivors, we explored response rates to study invitations formatted as greeting cards versus standard business letters. Survivors were identified from cancer registries and medical records and randomly assigned with strata defined by gender and racial/ethnicity to one-of-the-two invitations. Both groups received telephone follow-up.

**Results:**

Contact was verified among 708 survivors with an average age of 72 years with most being non-Hispanic White (NHW), urban dwelling, and female. Survivors assigned to the business letter (*n* = 360) as compared to the greeting card (*n* = 348) were significantly more likely to express interest in participation (OR 1.73, 95% CI 1.11–2.70). With the exception of racial/ethnic minorities (OR 0.73; 95% CI 0.26–2.11), all other subgroups favored the business letter with significance observed in females (OR 1.66, 95% CI 1.00–2.74), NHWs (OR 2.12; 95% CI 1.29–3.49), and rural dwellers (OR 3.61; 95% CI 1.49–8.76). Moreover, the business letter costs were substantially lower than the card.

**Conclusion:**

Clinical trial recruitment is significantly more effective if solicitations are formatted as standard business letters as compared to greeting cards, though this may not generalize to racial/ethnic minorities where more research is warranted.

**Implications for Cancer Survivors:**

These study findings are not only valuable to researchers but also have the potential to improve recruitment and engagement of older cancer survivors in clinical trials.

**Clinical Trial Registration:**

Harvest for Health for in Older Cancer Survivors, ClinicalTrials.gov Identifier: NCT02985411

## Introduction

Recruitment is essential to the success of a clinical trial, yet it is challenging and expensive [1]. One of the leading reasons for clinical trials’ failure is under recruitment [2]. An analysis of the ClinicalTrials.gov database shows that 57% of clinical trials were terminated due to insufficient accrual [3]. A 2013 report by the Tufts Center for the Study of Drug Development reported that 11% of pharmaceutical clinical trials failed to recruit even one participant, and 37% did not meet their accrual target [4]. A particular challenge with clinical trials is recruiting older cancer survivors, who are a high research priority and a population group that is expected to grow by 60% in the next 20 years [5].

Recruitment cost is a notable expense for clinical trials where oftentimes a majority of funding relates to this activity [6, 7]. Many studies use paid media to recruit older adults; however, this is an expensive and non-targeted strategy to improve response rate [8]. Therefore, less costly recruitment strategies are needed to improve study quality (by assuring adequate statistical power) and to lower cost. Mail-based recruitment methods are considered feasible and a targeted cost-effective approach for accruing participants into clinical trials compared to other strategies, such as paid media or in-person, clinical-based means [9]. Patterson et al. [10] reported that mail-based methods in which directed mailings are informed by information provided by cancer registries are particularly efficient in recruiting individuals to trials aimed at cancer survivorship.

Mail-based recruitment has long been considered a reliable method for community-based recruitment [11]. Mail-based strategies generally rely on brochures and letters to target specific population groups for research studies. While research has shown that a mail-based approach for recruitment and follow-up is less costly compared to in-person methods, there still exist some inefficiencies [12]. To date, studies have used various mail-based strategies to improve response rate. A direct mail recruitment strategy for Hispanic adults reported that flyers with a personalized hand-signed note resulted in significantly higher response rates (7.8%) when compared to flyers alone (2.1%) [13]. Relatedly, and in a Cochrane review of 481 studies using mailed surveys, Edwards et al. found several factors that were significantly related to increased response rates, such as the display of a teaser on the envelope (i.e., a comment suggesting to participants that they may benefit if they open it) (odds ratio [OR] = 3.08; 95% confidence interval [CI] 1.27 to 7.44), university sponsorship (OR = 1.32; 95% CI 1.13 to 1.54), personalization (OR = 1.14; 95% CI 1.07 to 1.22), and first-class outward mailing (1.11; 95% CI 1.02 to 1.21) [14]. However, relatively few studies have evaluated different formats of mail-based strategies to enhance clinical trial participation. The objective of the current study was to compare response rates to business letter versus greeting card invitations used to recruit older cancer survivors into a randomized controlled clinical trial of a lifestyle intervention. Given that Edwards et al. [14] also found that attractive illustrations invoked response rates that were threefold higher, we hypothesized a greater response rate from individuals receiving the greeting card invitation versus those receiving a standard business letter.

## Methods and materials

This comparison of mailed-based methods was undertaken as part of the “Harvest for Health” trial, a National Cancer Institute–sponsored randomized control trial that studies the impact of a home-based vegetable gardening intervention on the health behaviors and physical functioning of older cancer survivors across Alabama. The overall protocol for this registered trial (NCT02985411) and the current sub-study were approved by the University of Alabama at Birmingham (UAB) Institutional Review Board [15]. Potential participants were identified through the Alabama State Cancer Registry and UAB’s i2b2 cancer database, ascertaining individuals with cancer diagnoses consistent with the eligibility criteria for the trial. Recruitment began with a mailed solicitation, followed by a minimum of six phone calls which commenced 2 weeks following the mailing. If, following six call attempts, the individual had not responded, they are categorized as “disinterested,” as were individuals who directly expressed this response. Decedents and individuals whose letters were returned as undeliverable or who had non-working telephone numbers were identified as “uncontactable.”

**Fig. 1:**
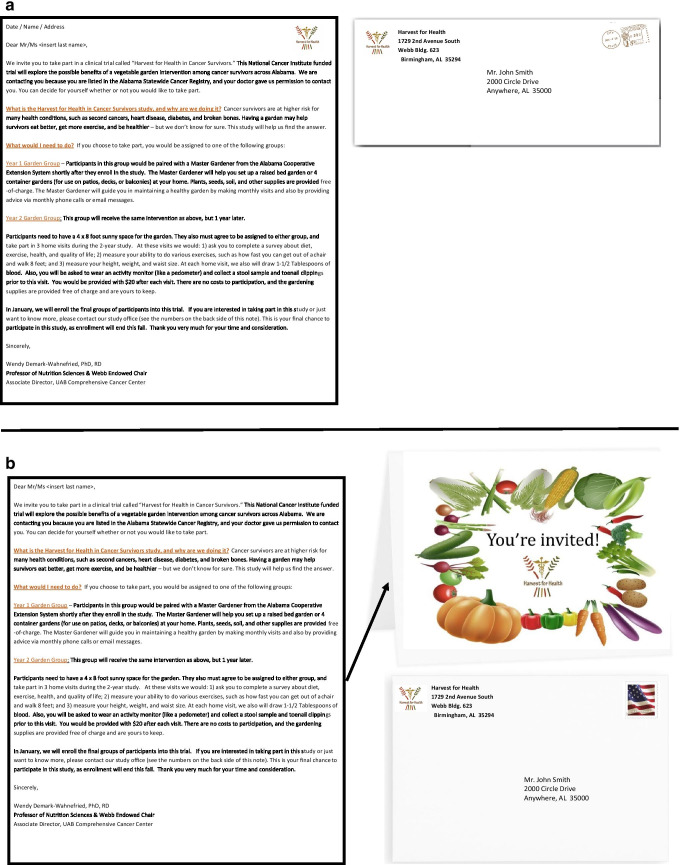
Illustrations of the standard business invitation (**a**) and the greeting card invitation (**b**)

The sub-study was undertaken during recruitment for the eleventh study cohort and occurred between July 2019 and November 2019, which was directed toward survivors residing in northern and central Alabama. Potential participants (*n* = 1150) were stratified by gender and minority status as previous findings have shown variation in response by these two factors in recruitment for lifestyle interventions [16, 17]. Individuals were randomized to receive one of the two document types: (1) business-style letter or (2) greeting card invitation. The business-style letter, used for recruitment in previous cohorts, was printed in black and white, with colored logo and headers, on 8.5″ by 11″ printer paper and was mailed in a 4 1/8″ by 9 1/2″, white, business-style envelope (Fig. 1a). The cost to produce the business style letter was US$0.13 in supply costs and approximately US$0.26 per letter in labor for a total per letter cost of US$0.39. The greeting card invitation was printed on 8.5″ by 11″ cardstock and folded in half. The card face featured a color image of a harvest basket of vegetables with the message “You’re Invited!” (Fig. 1b). The invitation card was mailed in a cream-colored, 4.75″ by 6.5″ envelope. The supply costs to produce the greeting card were US$0.51 cents, and labor costs were approximately US$0.49 cents per letter for a total production cost of US$1.00 per letter. The text inside the card was identical to the standard recruitment letter. Both mailings had the same address label, return address label, and included a bright green Harvest for Health brochure and a packet of 4–10 vegetable seeds. Both sets of mailings used first class US postage stamps; the rates of postage were identical for both, i.e., 55 cents. Likewise, the protocol for follow-up telephone calls was identical for both mailing groups. Labor costs included costs for conducting mail merges, printing, mailing assembly, affixing stamps, and transport to the post office.

Statistical analyses were performed using SAS v.9.4 (Cary, NC). Primary analysis was performed using a calculation of the odds ratio and 95% confidence interval of responding positively versus negatively, based on the type of invitation received. For the purposes of this analysis, a “positive” response was defined as expression of interest in participating in the study, whereas a negative response was either expressed disinterest in the study or a failure to make contact despite repeated calls. Analyses also were performed for each of the subgroups based on gender (men versus women) and race/ethnicity (non-Hispanic Whites (NHW) versus others). Given that data were available on whether individuals resided in counties that were categorized as either rural or urban as defined by both the Office of Primary Care and Rural Health within the Alabama Department of Public Health and the Alabama Rural Health Association [18], a comparison of rural versus urban county residents also was performed. Chi-square tests were conducted to confirm significance of proportions of individuals among stratified groups in the cohort. While exploratory, an alpha level of 0.05 was used to determine significant difference between mailing groups and afforded power of at least 80%.

## Results

Among this older population of survivors whose cancer diagnosis could be up to 5 years in the past, there were a significant number of individuals who could not be contacted as demonstrated by either letters returned as undeliverable or inactive phone numbers. Thus, of the 1150 letters that were mailed, contact could only be verified among 708 (~ 62%). Table 1 provides data on the characteristics of the cancer survivors for whom we confirmed contact by mailing group assignment. The overall study sample was roughly 84% NHW, 68% urban, 59% female, and had an average age of 72 years. No significant differences were detected between mailing groups with respect to age, minority status, gender, or rural/urban residence.

**Table 1:** Characteristics of cancer survivors receiving business letter vs. greeting card invitations

	Recruitment mailing style	
	Business letter (*n* = 360)	Greeting card (*n* = 348)
Age (mean years, SE)*	71.87 (0.42)	71.70 (0.46)
Gender (*N*, %)		
Male	152 (42.2%)	136 (39.1%)
Female	208 (57.8%)	212 (60.9%)
Race/ethnicity (*N*, %)		
Non-Hispanic White	309 (85.8%)	292 (83.9%)
Minority	51 (14.2%)	56 (16.1%)
Rural/urban county of residence (*N*, %)		
Rural	120 (33.3%)	108 (31.7%)
Urban	240 (66.7%)	240 (68.3%)

^*^Age unknown for 31 respondents

Table 2 shows responses to mailing type in the overall sample, as well as subgroups defined by minority status, gender, and place of residence. Overall, 13.6% of the cancer survivors contacted about the trial indicated an interest in participation. In general, higher positive response rates were noted with the business letter as compared to greeting card formatted invitations. These increased odds with the business letter were significant in the total sample (73% greater), as well as in females (66% greater), NHWs (roughly twofold greater), and among survivors residing in rural counties (more than threefold greater). The only subgroup that seemed to have reduced odds of a positive response to the business letter invitation was survivors of racial/ethnic minority groups; however, here, the difference in response rate was not significant.

**Table 2:** Responses to business letter versus greeting card invitations

	Business letter		Greeting card		OR (95% CI)
	Positive	Negative	Positive	Negative	
Overall — *N* (%)	60 (16.7%)	300 (83.7%)	36 (10.3%)	312 (89.7%)	**1.73 (1.11–2.70)**
Gender — *N* (%)					
Male	14 (9.2%)	138 (90.8%)	5 (3.7%)	131 (96.3%)	2.66 (0.93**–**7.56)
Female	46 (22.1%)	162 (77.9%)	31 (14.6%)	181 (85.4%)	**1.67 (1.00–2.74)**
Race/ethnicity— *N* (%)					
NHW	53 (17.2%)	256 (82.8%)	26 (10.3%)	266 (89.7%)	**2.12 (1.29–3.49)**
Minority	7 (14.3%)	42 (85.7%)	10 (18.5%)	44 (81.5%)	0.73 (0.26**–**2.11)
County of residence—*N* (%)					
Urban	36 (15.0%)	204 (85.0%)	29 (12.1%)	211 (87.9%)	1.28 (0.76**–**2.17)
Rural	24 (20.0%)	96 (80.0%)	7 (6.5%)	101 (93.5%)	**3.61 (1.49–8.76)**

Bold text depicts significant associations

## Discussion

Few studies have been conducted either among older adults or cancer survivors that have compared recruitment response rates by mailing style. Thus, this study adds to the knowledge base surrounding clinical trial accrual in this unique, but rapidly expanding patient population. Contrary to our hypothesis, standard business-style letters as compared to greeting card–formatted solicitations resulted in significantly greater recruitment success, not only in the overall sample, but in NHW, female, and rural subgroups as well. Given the higher response rates found with business letters, coupled with their much lower cost, the results of this study provide clear evidence that invitations to clinical trials should employ traditional mailing approaches. Potential explanations as to why more individuals may have responded to the business-style letter could be due to greater perceived trust, legitimacy, or urgency or belief that the letter contained medically pertinent information [19]. In a review that explores optimal strategies to invoke diet and exercise change among cancer survivors, Hoedjes and colleagues cite the credible source among the most frequently used methods [20]. It also bears noting our hypothesis favoring the greeting card invitation was largely based on the findings of Edwards et al., who noted that attractive illustrations bolstered response rates by 3.44, although the 95% confidence interval was quite broad, i.e., 0.72–16.4 [14]. Moreover, in this extensive review, and counter intuitively, neither higher quality paper nor larger envelope size resulted in higher response rates.

It is interesting that the business letter appeared to have less success among minority survivors and that the directionality of effect was opposite of the overall sample and all other subgroups. While not statistically significant (likely due to small numbers), this finding resonates with the research by Smirnoff and colleagues which suggests that the business letter may invoke a higher level of distrust which has been reported amongst the minority community. Creating trust and open and clear communication about expectations is a key factor reported in previous studies that have focused on recruitment [21]. Additionally, although letter content was not the focus of our study, other findings have suggested that mailings that include associated health risk information that is specific to a particular minority group, rather than general population risk information, significantly improves response rates [22]. As “surface-level” cues, targeted statements acknowledging ethnicity and associated health risks suggest that receivers can expect to find that study staff and environments potentially match their own ethnicities and health risk concerns [23, 24]. While our letter included references to increased risk of cancers and comorbid conditions within the general cancer survivor population, it did not contain minority-specific health risk information. Given that minority accrual to clinical trials continues to be a substantial challenge, additional studies on specific, targeted mailing formats and content that are amply powered may be warranted among racial/ethnic minorities.

While this study detected several findings of significance, the smaller sample size coupled with the poor response rate poses a limitation. However, it should be noted that the overall response rate of 13.6% for this randomized controlled trial is comparable to the 11% response rate noted for the Reach-Out to Enhance Wellness among Older Cancer Survivors trial that also targeted a similar population and required a 2-year commitment [25]. The proportion of uncontactable cancer survivors was certainly higher than anticipated, and while death and movement to senior care facilities is expected in older adult populations with significant comorbidities, factors such as changing cell phone numbers also likely played a role. Additional potential limitations include mailing delays and delivery errors, particularly among the rural population.

In conclusion, the results of this study largely support the use of standard business-style letters as compared to greeting card invitations as means to notify older cancer survivors of clinical trials. Not only do standard letters produce superior response rates, they are also less expensive. The only population that does not seem to demonstrate higher response rates to standard letter formats is minority cancer survivors. Further and more expansive research that explores mailing type may be warranted in this population.

## Data Availability

The datasets generated during and/or analyzed during the current study are available from the corresponding author on reasonable request.

## Code availability

N/A

## References

[1] McDonald AM, Knight RC, Campbell MK, Entwistle VA, Grant AM, Cook JA (2006). What influences recruitment to randomized controlled trials? A review of trials funded by two UK funding agencies. Trials.

[2] Desai M (2020). Recruitment and retention of participants in clinical studies: critical issues and challenges. Perspect Clin Res.

[3] Williams RJ, Tse T, DiPiazza K, Zarin DA. Terminated trials in the ClinicalTrials.gov Results database: evaluation of availability of primary outcome data and reasons for termination. PLoS One. 2015. 10.1371/journal.pone.0127242. 10.1371/journal.pone.0127242 PMC4444136 26011295

[4] Tufts University Center for the Study of Drug Development. 89% of trials meet enrollment, but timelines slip, half of sites under-enroll. Impact Report 2013;15(1). https://static1.squarespace.com/static/5a9eb0c8e2ccd1158288d8dc/t/5aa2c28fec212d492f36cc8a/1520616079359/Jan-Feb+2013+IR+summary.pdf. Accessed online 03.01.2021

[5] Sidney S, Go AS, Jaffe MG, Solomon MD, Ambrosy AP, Rana JS. Association between aging of the US population and heart disease mortality From 2011 to 2017. JAMA Cardiol. 2019.10.1001/jamacardio.2019.4187 10.1001/jamacardio.2019.4187 PMC6822092 31663094

[6] Gismondi PM, Hamer DH, Leka LS, Dallal G, Fiatarone Singh MA (2005). Strategies, time, and costs associated with the recruitment and enrollment of nursing home residents for a micronutrient supplementation clinical trial. J Gerontol A Biol Sci Med Sci.

[7] Chin Feman SP, Nguyen LT, Quilty MT, Kerr CE, Nam BH, Conboy LA (2008). Effectiveness of recruitment in clinical trials: an analysis of methods used in a trial for irritable bowel syndrome patients. Contemp Clin Trials.

[8] Kakumanu S, Manns BJ, Tran S, Saunders-Smith T, Hemmelgarn BR, Tonelli M (2019). Cost analysis and efficacy of recruitment strategies used in a large pragmatic community-based clinical trial targeting low-income seniors: a comparative descriptive analysis. Trials.

[9] Jurascheck SP, Plante TB, Charleston J, Miller ER, Yeh H, Appel LJ, et al. Use of online recruitment strategies in a randomized trial of cancer survivors. Clinical Trials (London, England). 2018. 10.1177/1740774517745829. 10.1177/1740774517745829 PMC5891380 29361843

[10] Patterson RE, Marinac CR, Natarajan L, Hartman SJ, Cadmus-Bertram L, Flatt SW (2016). Recruitment strategies, design, and participant characteristics in a trial of weight-loss and metformin in breast cancer survivors. Contemp Clin Trials.

[11] Lovato LC, Hill K, Hertert S, Hunninghake DB, Probstfield JL (1997). Recruitment for controlled clinical trials: literature summary and annotated bibliography. Control Clin Trials.

[12] Tworoger SS, Yasui Y, Ulrich CM, Nakamura K, LaCroix K, Johnston R (2002). Mailing strategies and recruitment into an intervention trial of the exercise effect on breast cancer survivors. Cancer Epidemiol Biomarkers Prev.

[13] Kiernan M, Phillips K, Fair JM, King AC (2000). Using direct mail to recruit Hispanic adults into a dietary intervention: an experimental study 1, 2, 3. Ann Behav Med.

[14] Edwards PJ, Roberts I, Clarke MJ, Diguiseppi C, Wentz R, Kwan I (2009). Methods to increase response to postal and electronic questionnaires. Cochrane Database Syst Rev.

[15] Bail JR, Frugé AD, Cases MG, De Los Santos JF, Locher JL, Smith KP (2018). A home-based mentored vegetable gardening intervention demonstrates feasibility and improvements in physical activity and performance among breast cancer survivors. Cancer.

[16] Marsh AP, Lovato LC, Glynn NW, Kennedy K, Castro C, Domanchuk K, PA. Lifestyle Interventions and Independence for Elders Study: recruitment and baseline characteristics. J Gerontol. 2013. 10.1093/gerona/glt064. 10.1093/gerona/glt064 PMC3814232 23716501

[17] Estabrooks P, You W, Hedrick V, Reinholt M, Dohm E, Zoellner J (2017). A pragmatic examination of active and passive recruitment methods to improve the reach of community lifestyle programs: the Talking Health Trial. Int J Behav Nutr Phys.

[18] Alabama Rural Health Association. “Definition of rural Alabama.” Alabama Rural Health Association. 2018. https://arhaonline.org/definition-of-rural-alabama/ Accessed online 03.01.2021.

[19] Guillemin M, Barnard E, Allen A, Stewart P, Walker H, Rosenthal D (2018). Do research participants trust researchers or their institution?. J Empir Res Hum Res Ethics.

[20] Hoedjes M, van Stralen MM, Joe STA, Rookus M, van Leeuwen F, Michie S, Seidell JC, Kampman E (2017). Toward the optimal strategy for sustained weight loss in overweight cancer survivors: a systematic review of the literature. J Cancer Surviv.

[21] Smirnoff M, Wilets I, Ragin DF, Adams R, Holohan J, Rhodes R (2018). A paradigm for understanding trust and mistrust in medical research: the Community VOICES study. AJOB Empir Bioeth.

[22] Brown, SD, Lee, K., Schoffman, BA, King, ACl, Crawley, LM, Kierman, M. Minority recruitment into clinical trials: experimental findings and practical implications. Contemp Clin Trials. 2012. 10.1016/j.cct.2012.03.003. 10.1016/j.cct.2012.03.003 PMC3361553 22449836

[23] Kiernan M, Edwards K, Fair JM, King AC (2000). Using direct mail to recruit Hispanic adults into a dietary intervention: An experimental study. Ann Behav Med.

[24] Caldwell PH, Hamilton S, Tan A, Craig JC (2010). Strategies for increasing recruitment to randomised controlled trials: systematic review. PLoS Med.

[25] Snyder DC, Morey MC, Sloane R, Stull V, Cohen HJ, Peterson B (2009). Reach out to ENhancE Wellness in Older Cancer Survivors (RENEW): design, methods and recruitment challenges of a home-based exercise and diet intervention to improve physical function among long-term survivors of breast, prostate, and colorectal cancer. Psychooncol.

